# Successful resection of a sessile serrated lesion completely involving a colonic diverticulum by endoscopic submucosal dissection with water pressure method

**DOI:** 10.1055/a-2218-3103

**Published:** 2024-01-09

**Authors:** Takahiro Matsunaga, Seiichiro Fukuhara, Motoki Sasaki, Motohiko Kato

**Affiliations:** 1 Department of Gastroenterology and Hepatology, National Hospital Organization Tokyo Medical Center, Tokyo, Japan; 2 Division of Research and Development for Minimally Invasive Treatment, Cancer Center, Keio University School of Medicine, Tokyo, Japan; 3 Center for Diagnostic and Therapeutic Endoscopy, Keio University School of Medicine, Tokyo, Japan

Endoscopic submucosal dissection (ESD) for lesions involving colonic diverticula is technically challenging owing to lack of a proper muscle layer. Herein, we describe successful resection of a colonic lesion, which extended along the entire length of a diverticulum, using ESD with water pressure method ( Video 1 ); this method is used in highly difficult duodenal ESD 1 .

Successful resection of a sessile serrated lesion, which extended along the full length of a colonic diverticulum, using endoscopic submucosal dissection with water pressure method.Video 1

A 77-year-old man was referred to our hospital because of a positive fecal occult blood test. Colonoscopy revealed a 15-mm Paris type 0-IIa sessile serrated lesion (SSL) in the cecum ( Fig. 1 ). The lesion extended along the entire length of a diverticulum on the left side, with two additional diverticula located on the anal side and oral left side of the lesion.

**Fig. 1:**
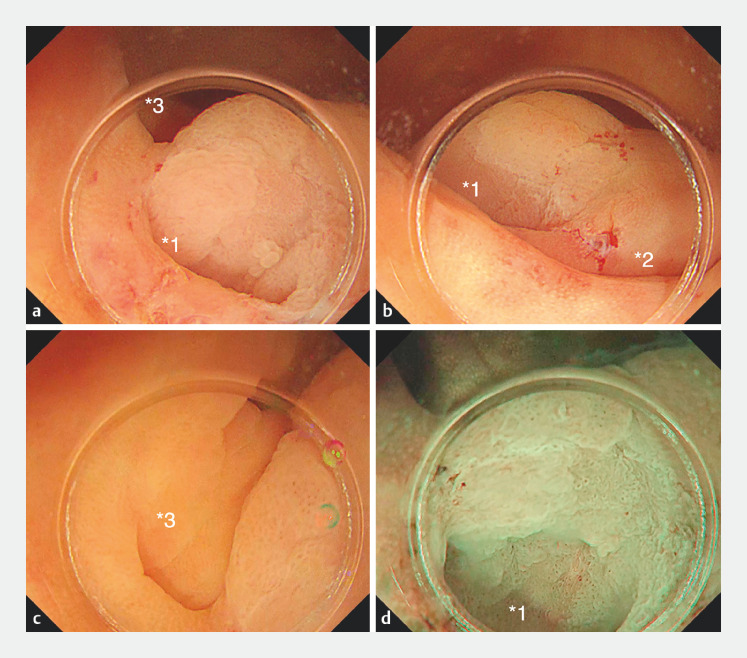
Endoscopy images. **a–c** The lesion was in the cecum, surrounded by three diverticula (*1, left side; *2, anal side; *3, oral left side). **d** The lesion extended along the entire length of the diverticulum on the left side (*1).

Air in the working space was aspirated and the lumen was filled with normal saline. Mucosal incision and trimming were performed from the anal side of the lesion and extending into the diverticulum on the left side of the lesion ( Fig. 2 **a** ). Submucosal dissection was performed using waterjet pressure ( Fig. 2 **b** ). A defect in the proper muscle layer was detected during dissection in the diverticulum ( Fig. 2 **c** ); we continued dissection, taking care not to cause full-thickness perforation ( Fig. 2 **d** ). After lesion dissection in the diverticulum, mucosal incision and trimming were performed on the oral side of the lesion while continuing dissection on the anal side. The lesion was resected en bloc, including the full length of the diverticulum, without perforation ( Fig. 2 **e,f,g** ). The wound was closed using endoscopic clips ( Fig. 2 **h** ). Pathology revealed SSL and complete resection of the tumor.

**Fig. 2:**
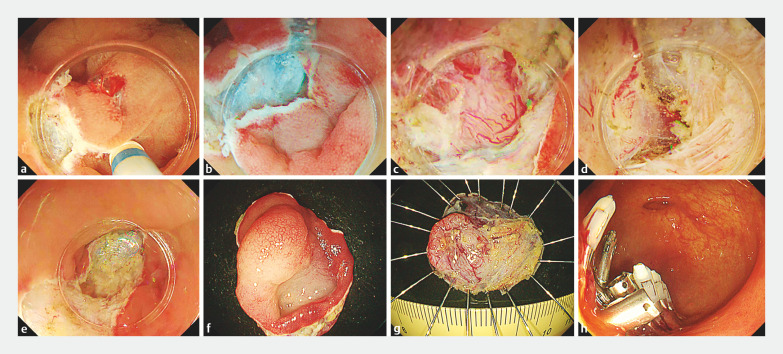
Endoscopic submucosal dissection procedure. **a** Mucosal incision and trimming were started on the anal side of the lesion. **b** Submucosal dissection of the anal side of the lesion. **c** A defect in the proper muscle layer was detected. **d** Dissection of the lesion continued into the diverticulum on the left side of the lesion. **e–g** The lesion was resected en bloc, including from the diverticulum on the left side. **h** The wound was completely closed using endoscopic clips.

Under air insufflation, a diverticulum expands due to air pressure, making effective submucosal injection difficult, and increasing the angle of the endoscope and the risk of perforation ( Fig. 3 **a** ). In contrast, under water pressure conditions reduce the tension in the diverticulum, making effective submucosal injection possible and submucosal dissection easier with the help of buoyancy ( Fig. 3 **b** ) 2 . Low intraluminal pressure also helps to avoid perforation.

**Fig. 3:**
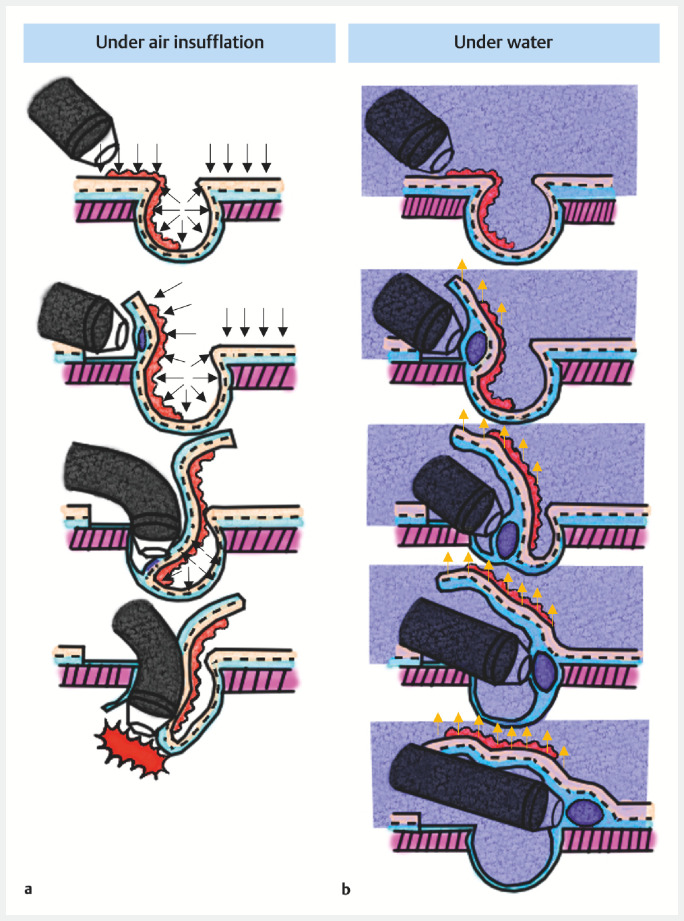
Schematic diagrams of endoscopic submucosal dissection for a lesion involving a diverticulum. **a** Under air insufflation, the diverticulum expands due to air pressure, making effective submucosal injection difficult, and increasing the angle of the endoscope and the risk of perforation. **b** Under water pressure conditions, the tension in the diverticulum is reduced, making effective submucosal injection possible and submucosal dissection easier with the help of buoyancy. Low intraluminal pressure also helps to avoid perforation.

Endoscopy_UCTN_Code_TTT_1AQ_2AD

## Notes

Endoscopy E-Videos https://eref.thieme.de/e-videos
*E-Videos* is an open access online section of the journal *Endoscopy* , reporting on interesting cases and new techniques in gastroenterological endoscopy. All papers include a high-quality video and are published with a Creative Commons CC-BY license. Endoscopy E-Videos qualify for HINARI discounts and waivers and eligibility is automatically checked during the submission process. We grant 100% waivers to articles whose corresponding authors are based in Group A countries and 50% waivers to those who are based in Group B countries as classified by Research4Life (see: https://www.research4life.org/access/eligibility/ ). This section has its own submission website at https://mc.manuscriptcentral.com/e-videos .
